# Crystal structure of CaSiF_6_·2H_2_O(*mP*2) and reevaluation of the Si^IV^–F bond-valence parameter *R*_0_

**DOI:** 10.1107/S2056989023009349

**Published:** 2023-11-02

**Authors:** Klemen Motaln, Matic Lozinšek

**Affiliations:** a Jožef Stefan Institute, Jamova cesta 39, 1000 Ljubljana, Slovenia; b Jožef Stefan International Postgraduate School, Jamova cesta 39, 1000 Ljubljana, Slovenia; University of Aberdeen, United Kingdom

**Keywords:** calcium hexa­fluorido­silicate, bond-valence parameter, crystal structure, disorder, hydrogen bonding

## Abstract

The structure of a second polymorph of CaSiF_6_·2H_2_O [calcium hexafluorido­silicate dihydrate; space group *P*2/*c* (No. 13), Pearson symbol *mP*2] was elucidated by single-crystal X-ray diffraction. It arose as an unexpected product when soda-lime glass was attacked by HF. Its crystal structure consists of infinite _∞_^2^[Ca(H_2_O)_2/1_(SiF_6_)_4/4_] layers oriented parallel to the *bc*-crystallographic plane, a unique motif among structurally characterized hydrated hexa­fluorido­silicates. The crystal structure also exhibits inter- and intra­layer hydrogen bonds, with the inter­layer O—H⋯O hydrogen bonds involving a disordered hydrogen atom. The large deviation between the calculated bond-valence sum for Si and the expected value prompted a redetermination of the empirical Si^IV^–F bond-valence parameter *R*_0_. Based on a data set of 42 high-quality crystal structures containing 49 independent Si^IV^ coordination environments, a revised value of 1.534 Å was derived for *R*_0_.

## Abstract 2

The crystal structure of a second polymorph of CaSiF_6_·2H_2_O featuring a layered structure connected by hydrogen bonds is presented.

## 1. Chemical context

Calcium hexa­fluorido­silicate (CaSiF_6_) and its hydrated form, calcium hexa­fluorido­silicate dihydrate (CaSiF_6_·2H_2_O), are both commercially available chemicals that have found numerous uses, including as additives for cement manufacture (Smart & Roy, 1979 ▸), improving dentine remediation treatments (Kawasaki *et al.*, 1996 ▸), and as precursors for synthesis of luminescent materials (Kubus & Meyer, 2013 ▸). Although the synthesis of CaSiF_6_·2H_2_O and its dehydration to CaSiF_6_ were investigated more than 90 years ago (Carter, 1932 ▸), their crystal structures were determined relatively recently by laboratory-based powder X-ray diffraction using simulated annealing and Rietveld refinement (Frisoni *et al.*, 2011 ▸). The study revealed that CaSiF_6_·2H_2_O crystallizes in the monoclinic crystal system (space group *P*2_1_/*n*, Pearson symbol *mP*4) and exhibits a three-dimensional framework structure. In this work, the crystal structure of a second polymorph of CaSiF_6_·2H_2_O (space group *P*2/*c*, Pearson symbol *mP*2) was determined by low-temperature single-crystal X-ray diffraction. The observed discrepancies between the calculated and expected bond-valence sum (BVS) for Si also provided the impetus for a reevaluation of the Si^IV^–F bond-valence parameter *R*_0_ and an improved value of *R*_0_ was determined.

## 2. Structural commentary

**Figure 1:**
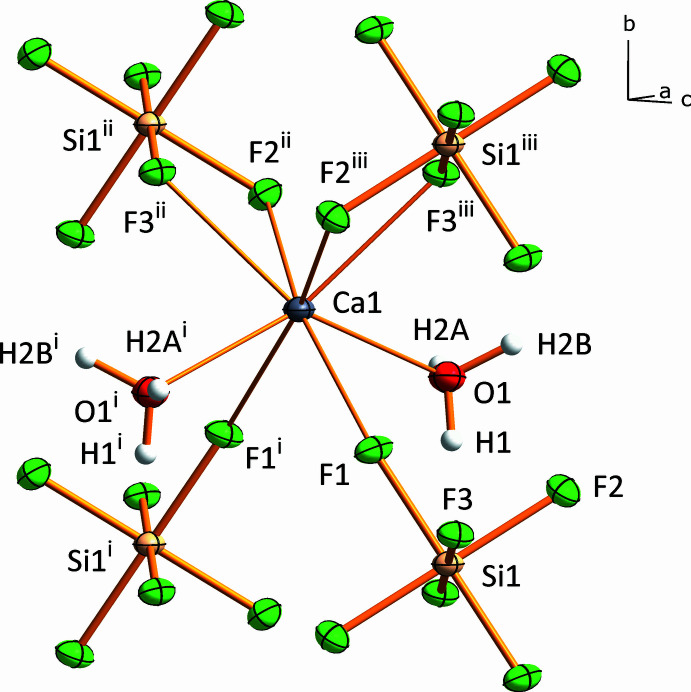
The distorted square anti­prismatic coordination environment of the Ca^2+^ cation in the crystal structure of CaSiF_6_·2H_2_O(*mP*2). Displacement ellipsoids are drawn at the 50% probability level and hydrogen atoms shown as spheres of arbitrary radius. Hydrogen atom H2 is disordered over two sites with occupancies 0.49 (5) and 0.51 (5) [Symmetry codes: (i) −*x*, *y*, −*z* + 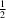 ; (ii) *x*, −*y* + 1, *z* − 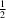 ; (iii) −*x*, −*y* + 1, −*z* + 1.]

**Figure 2:**
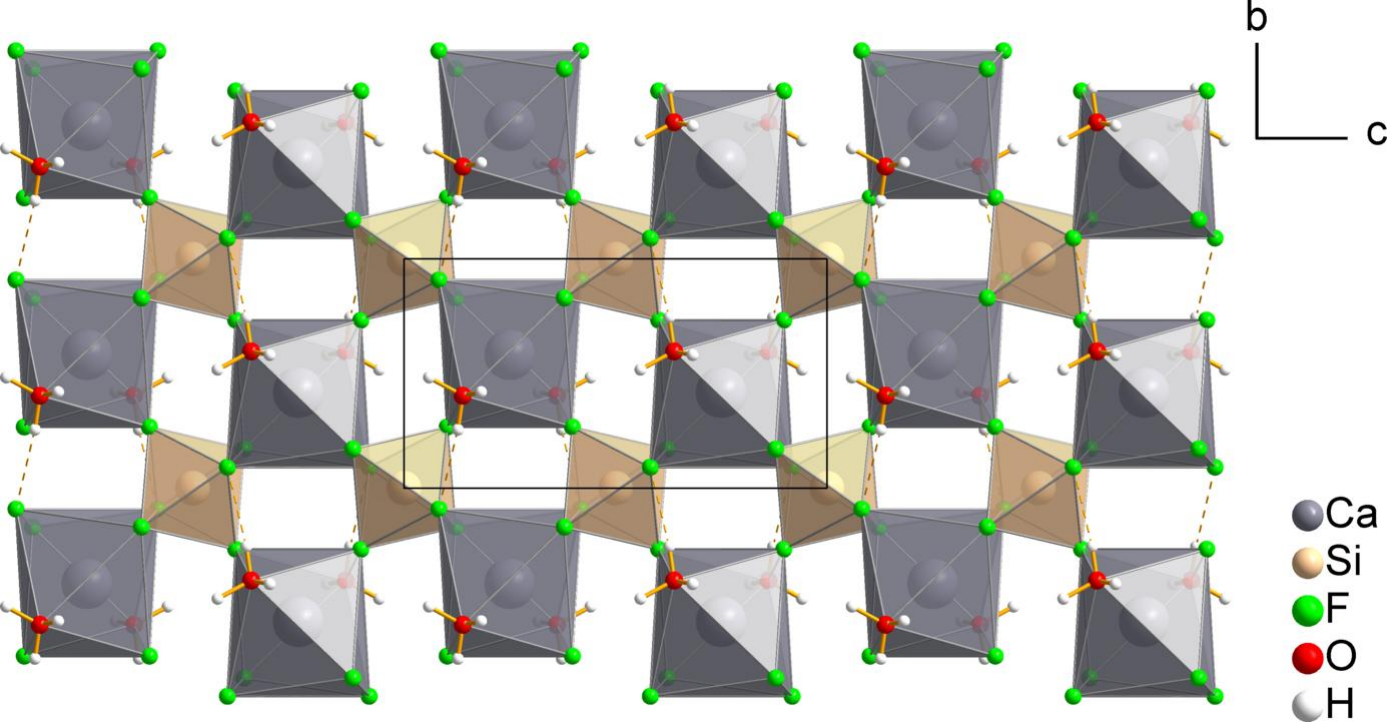
A single _∞_^2^[Ca(H_2_O)_2/1_(SiF_6_)_4/4_] layer viewed along [100], with the intra­layer O—H⋯F hydrogen bonds depicted as dashed lines.

The crystal structure of CaSiF_6_·2H_2_O(*mP*2) features eight atoms in the asymmetric unit, with one hydrogen atom disordered over two positions. The Ca atom is located on a twofold rotation axis and the Si atom is situated on an inversion centre, whereas the light atoms all lie on general positions. The hexa­fluorido­silicate anion displays a nearly ideal octa­hedral coordination, with the *cis*-F—Si—F angles ranging from 88.37 (4) to 91.63 (4)°. The average Si—F bond length is 1.6859 Å (Table 1 ▸), with the bond lengths ranging from 1.6808 (9) to 1.6942 (9) Å, which is in good agreement with the Si—F distances observed in the crystal structures of CaSiF_6_·2H_2_O(*mP*4) (Frisoni *et al.*, 2011 ▸) and SrSiF_6_·2H_2_O (Golovastikov & Belov, 1982 ▸), which span from 1.648 (4) to 1.701 (3) Å and 1.675 (5) to 1.700 (5) Å, respectively. The Ca atom is coordinated by six fluorine atoms at distances of 2.2965 (9)–2.4105 (9) Å originating from four neighbouring [SiF_6_]^2–^ octa­hedra, two of which are bound to the metal centre in a bidentate and two in a monodentate manner. In turn, each [SiF_6_]^2–^ octa­hedron is coordinated to four Ca^2+^ cations. The primary coordination sphere of the Ca^2+^ cation is completed by two water mol­ecules, with a Ca—O distance of 2.4331 (13) Å, resulting in a distorted square anti­prismatic coordination (Fig. 1 ▸). Such connectivity leads to the formation of _∞_^2^[Ca(H_2_O)_2/1_(SiF_6_)_4/4_] (Jensen, 1989 ▸) infinite layers, which extend along the *bc*-crystallographic plane and are stacked along the *a*-axis (Fig. 2 ▸), a structural motif that differs from all other hydrated hexa­fluorido­silicates. Bond-valence sum calculations (Brown, 2009 ▸) for Ca and Si using the parameters *b* = 0.37, *R*_0_ = 1.842 Å (Ca–F), *R*_0_ = 1.967 Å (Ca–O), and *R*_0_ = 1.58 Å (Si–F) obtained from the literature (Brown 2020 ▸; Brown & Altermatt, 1985 ▸; Brese & O’Keeffe, 1991 ▸), yielded 2.05 valence units (v.u.) for Ca and 4.51 v.u. for Si (expected values: 2 for Ca, 4 for Si). Similarly inflated values for the bond-valence sum of Si were also observed when other crystal structures of hexa­fluorido­silicates were examined, indicating the need to reevaluate the current Si^IV^–F parameter *R*_0_ (Section 5).

## 3. Supra­molecular features

**Figure 3:**
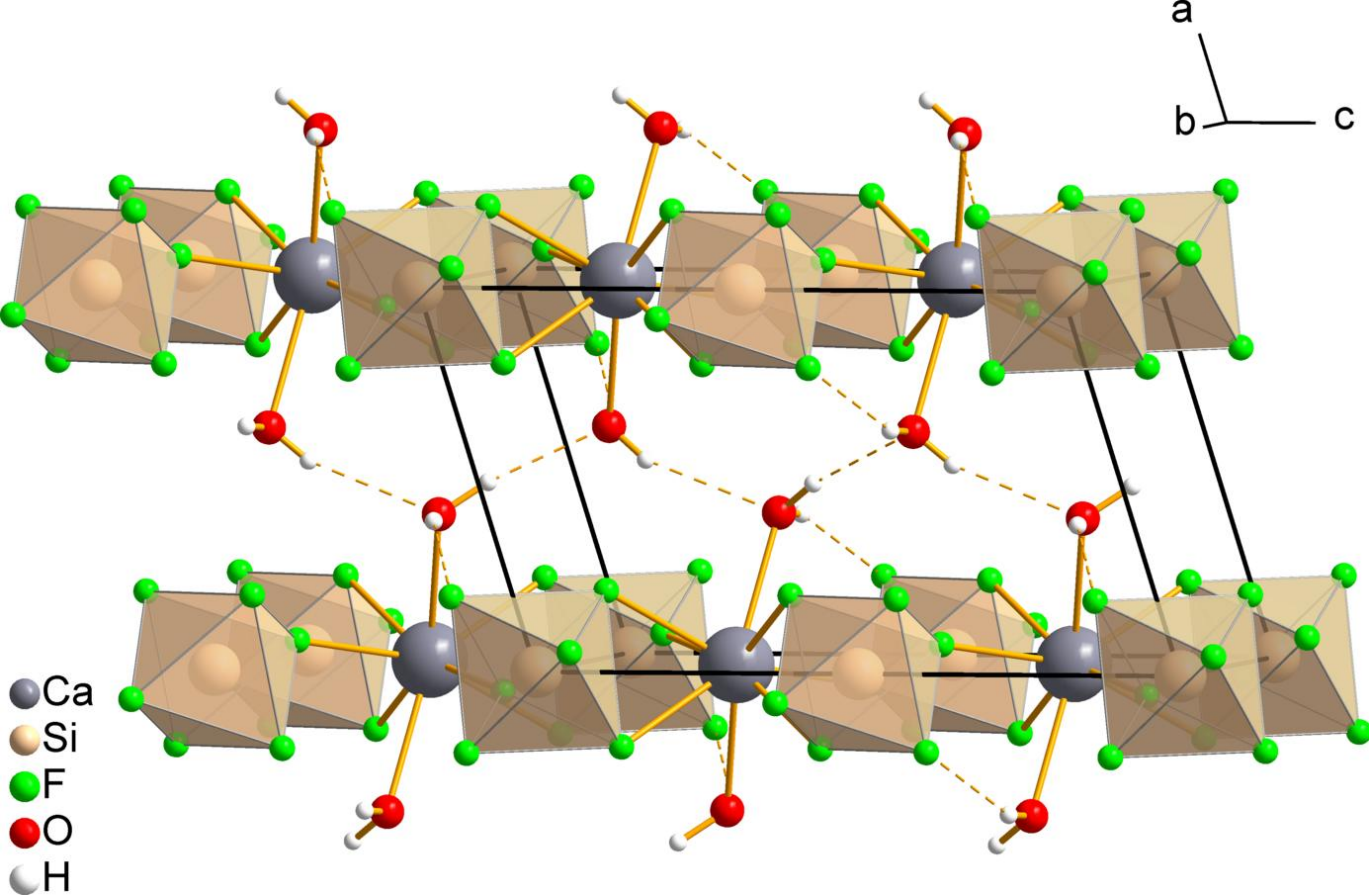
Selected fragment of the crystal structure of CaSiF_6_·2H_2_O(*mP*2) displaying intra- and inter­layer hydrogen bonds, which connect the adjacent layers. Some of the disordered hydrogen atoms have been omitted for clarity.

The crystal structure of CaSiF_6_·2H_2_O(*mP*2) exhibits both intra­layer O—H⋯F and inter­layer O—H⋯O hydrogen bonds (Table 2 ▸, Fig. 3 ▸). The intra­layer hydrogen bonds are formed between the F3 atom and the non-disordered hydrogen atom H1, with an O1⋯F3 distance of 2.9042 (14) Å and a graph-set motif of *S*(6) (Etter *et al.*, 1990 ▸). The oxygen atom O1 is involved in two further hydrogen bonds with the disordered hydrogen atoms H2*A* and H2*B*, forming O1—H2*A*⋯O1 and O1—H2*B*⋯O1 hydrogen bonds, with O⋯O distances of 2.902 (3) and 2.856 (3) Å, respectively, that link the adjacent _∞_^2^[Ca(H_2_O)_2/1_(SiF_6_)_4/4_] layers.

## 4. Database survey

**Figure 4:**
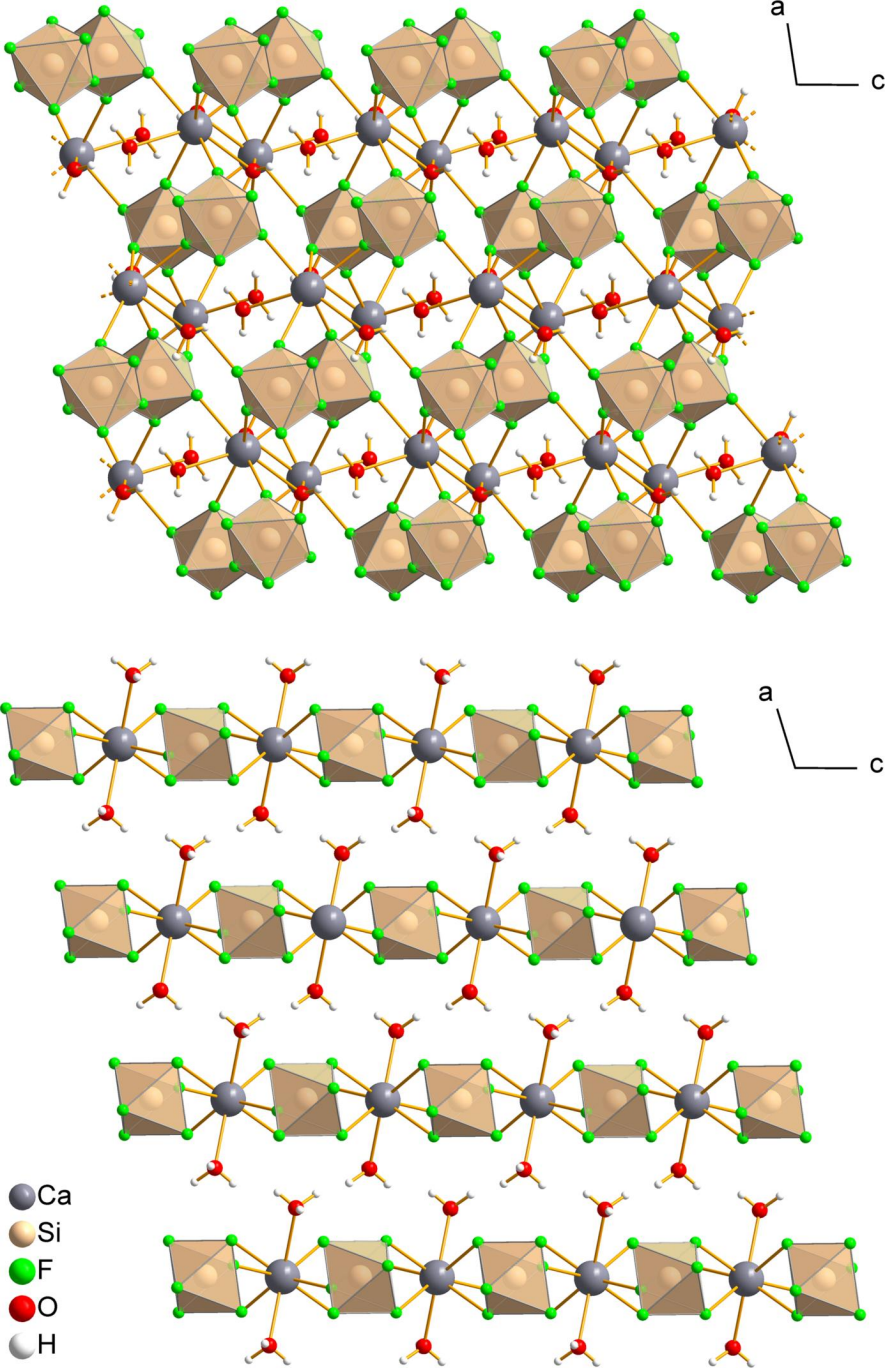
Comparison of the crystal structures of CaSiF_6_·2H_2_O(*mP*4) (top) and CaSiF_6_·2H_2_O(*mP*2) (bottom), viewed along [010].

A search of the Inorganic Crystal Structure Database (ICSD, version January 2023; Bergerhoff *et al.*, 1983 ▸; Zagorac *et al.*, 2019 ▸) revealed that in addition to the aforementioned *mP*4 polymorph of CaSiF_6_·2H_2_O, twelve other hydrated hexa­fluorido­silicates of divalent cations have been crystallographically characterized to date. Most of them form hexa­hydrates with the general formula *M*SiF_6_·6H_2_O, where *M* = Mg (Syoyama & Osaki, 1972 ▸; Cherkasova *et al.*, 2004 ▸), Cr (Cotton *et al.*, 1992 ▸), Mn (Torii *et al.*, 1997 ▸), Fe (Hamilton, 1962 ▸; Chevrier *et al.*, 1981 ▸), Co (Lynton & Siew; 1973 ▸; Ray *et al.*, 1973*a* ▸; Ray & Mostafa, 1996 ▸), Ni (Ray *et al.*, 1973*a* ▸), Cu (Ray *et al.*, 1973*b* ▸), and Zn (Ray *et al.*, 1973*a* ▸). The aforementioned compounds all exhibit a similar structural motif composed of alternating discrete [*M*(H_2_O)_6_]^2+^ and [SiF_6_]^2–^ octa­hedra, connected *via* O—H⋯F hydrogen bonds into a three-dimensional network. The only examples of tetra­hydrated metal(II) hexa­fluorido­silicates are the isostructural CrSiF_6_·4H_2_O (Cotton *et al.*, 1993 ▸) and CuSiF_6_·4H_2_O (Clark *et al.*, 1969 ▸; Schnering & Vu, 1983 ▸; Troyanov *et al.*, 1992 ▸; Cotton *et al.*, 1993 ▸). In their crystal structures, infinite zigzag chains are formed by the coordination of two [SiF_6_]^2–^ octa­hedra to the apical positions of the square-planar [*M*(H_2_O)_4_]^2+^ units. The resulting highly distorted octa­hedral coordination surrounding the metal centre is characteristic of the Jahn–Teller active cations. The individual chains in the structures are connected by O—H⋯F hydrogen bonds that link the terminal fluorine atoms of the [SiF_6_]^2–^ units to the water mol­ecules coordinating the metal centres of the adjacent chains. Lastly there are three examples of metal(II) hexa­fluorido­silicate dihydrates, the isostructural pair CaSiF_6_·2H_2_O(*mP*4) (Frisoni *et al.*, 2011 ▸) and SrSiF_6_·2H_2_O (Golovastikov & Belov, 1982 ▸), and PbSiF_6_·2H_2_O (Golubev *et al.*, 1991 ▸). All three compounds feature an extended three-dimensional framework structure and display water mol­ecules bridging the metal centres, giving rise to dimeric [(H_2_O)*M*(μ-H_2_O)_2_ *M*(OH_2_)]^4+^ units for *M* = Ca, Sr and the more complex [Pb_4_(H_2_O)_6_]^8+^ units in the structure of PbSiF_6_·2H_2_O, which contain both μ- and μ_3_-water mol­ecules. The Ca^2+^ cation in CaSiF_6_·2H_2_O(*mP*4) is coordinated by five fluorine and three oxygen atoms arranged in a distorted square-anti­prismatic coordination. Each of the five fluorine atoms coordinated to the Ca^2+^ ion belongs to a separate [SiF_6_]^2–^ octa­hedron, which contrasts with the structure of the newly discovered *mP*2 polymorph, where both monodentate and bidentate coord­ination of the [SiF_6_]^2–^ anions to the Ca^2+^ cations is observed (Fig. 4 ▸). Conversely, each [SiF_6_]^2–^ anion in the structure of CaSiF_6_·2H_2_O(*mP*4) coordinates five neighbouring Ca^2+^ cations, leaving one terminal fluorine atom, which in turn accepts O—H⋯F hydrogen bonds from two water ligands.

## 5. Redetermination of Si^IV^–F bond-valence parameter *R*_0_

In order to determine a more accurate value of the Si^IV^–F bond-valence parameter *R*_0_, the ICSD was searched for all crystal structures containing Si^IV^ in an exclusively fluorine environment. To ensure that only high-quality data were used for the calculation of the *R*_0_ parameter, the data set was limited to crystal structures solved by single-crystal X-ray diffraction at ambient or low-temperature conditions, excluding disordered structures or those with an *R*_1_-value above 0.05. A data set of 42 crystal structures was obtained, containing a total of 49 independent Si^IV^ coordination environments, including the compound presented herein (Table 3 ▸). The *R*_0*i*_ value for each individual Si coordination environment was calculated using formula (A1.3) from the literature (Brown, 2002 ▸), which assumes a fixed value for the *b* parameter (0.37 Å). An improved value for the *R*_0_ parameter, 1.534 Å, was obtained by averaging the *R*_0*i*_ values, which ranged from 1.508 to 1.562 Å. BVS calculations employing the new empirical parameter yield significantly improved results compared to the calculations performed with the previously reported parameter, as 46 out of 49 evaluated coordination environments give a bond-valence sum within ±0.2 v.u. of the expected value (3.8–4.2 v.u.), in contrast to only a single one when using the old parameter (Table 4 ▸).

## 6. Synthesis and crystallization

Colourless single crystals of the title compound were discovered to have grown serendipitously on a soda-lime watch glass containing a sample of [XeF][SbF_6_] (Gillespie & Landa, 1973 ▸) frozen under a protective layer of perfluoro­deca­lin at 255 K. It is presumed that CaSiF_6_·2H_2_O(*mP*2) formed when the soda-lime glass was attacked by the HF forming during hydrolysis of the highly oxidizing Xe^II^ compound.

## 7. Raman spectroscopy

A Bruker Senterra II confocal Raman microscope was used to record the Raman spectrum on a randomly oriented single crystal of the title compound. The spectrum was measured at room temperature (297 K) in the 50–4250 cm^−1^ range with a resolution of 4 cm^−1^ using the 532 nm laser line operating at 12.5 mW.

**Figure 5:**
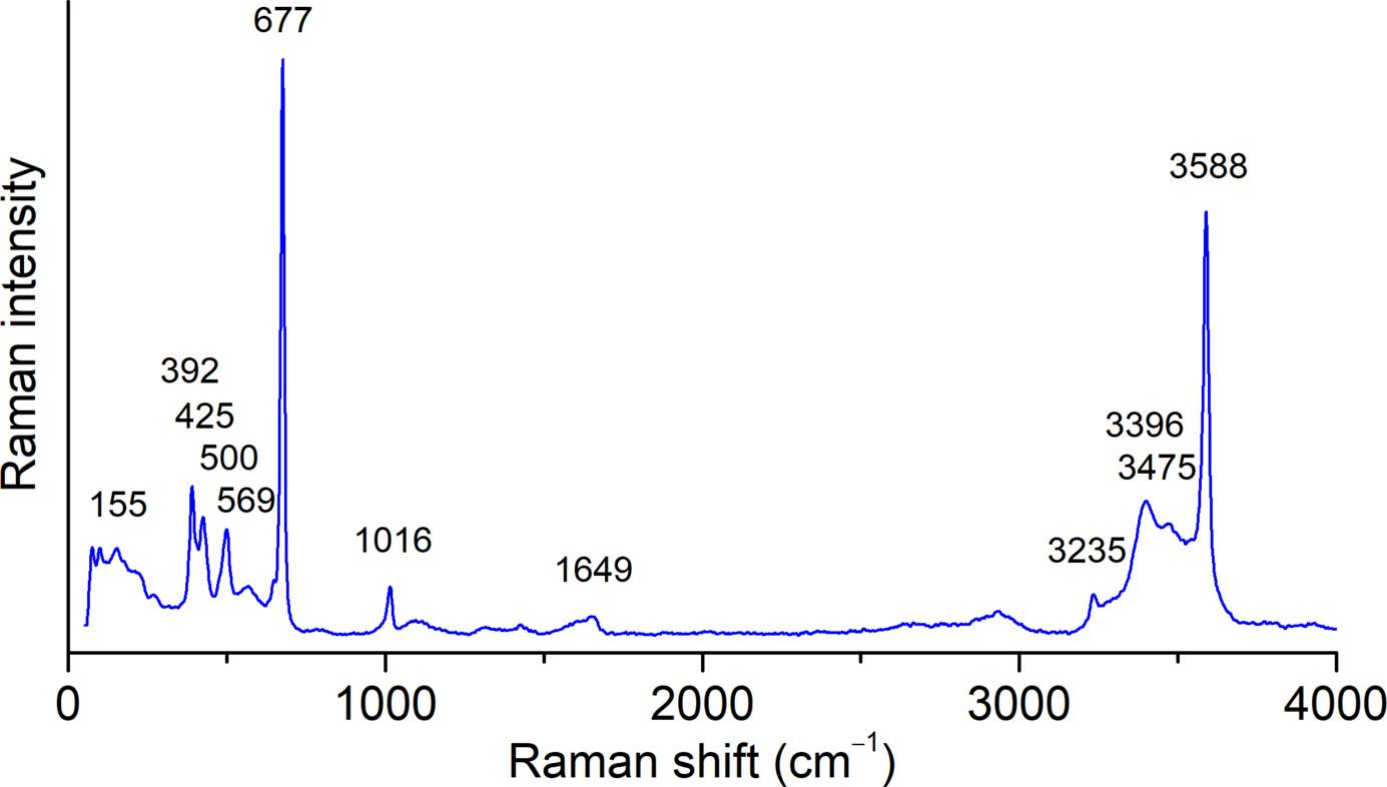
Raman spectrum of CaSiF_6_·2H_2_O(*mP*2).

In the Raman spectrum of CaSiF_6_·2H_2_O(*mP*2) (Fig. 5 ▸) the bands observed at 677 and 500 cm^−1^ correspond to the ν_1_ and ν_2_ modes of the [SiF_6_]^2–^ anion, respectively. The bands at 425 and 392 cm^−1^ can be assigned to the ν_5_ mode, split due to the distortion of the anion from the ideal *O*_h_ symmetry (Ouasri *et al.*, 2002 ▸). The Raman bands observed in the 3300–3600 cm^−1^ region belong to the symmetric ν_1_ and anti­symmetric ν_3_ O—H stretching of the coordinated water mol­ecules, whereas the bands at 1649 and 3225 cm^−1^ could likely be assigned to δ(HOH) (ν_2_) and 2δ(HOH), respectively (Lacroix *et al.*, 2018 ▸).

## 8. Refinement

Crystal data, data collection and structure refinement details are summarized in Table 5 ▸. The positions of the hydrogen atoms, including the disordered one, were located in difference maps and freely refined, including their isotropic thermal parameter *U*_iso_ (Cooper *et al.*, 2010 ▸). The refinement of the disordered hydrogen atoms’ occupancies, resulted in values of 0.51 (5) and 0.49 (5) for H2*A* and H2*B*, respectively.

## Supplementary Material

Crystal structure: contains datablock(s) I. DOI: 10.1107/S2056989023009349/hb8080sup1.cif

Structure factors: contains datablock(s) I. DOI: 10.1107/S2056989023009349/hb8080Isup2.hkl

CCDC reference: 2303630

Additional supporting information:  crystallographic information; 3D view; checkCIF report

## Figures and Tables

**Table 1:** Selected bond lengths (Å)

Ca1—F1	2.2965 (9)	Si1—F1	1.6809 (9)
Ca1—F2^i^	2.3783 (9)	Si1—F2	1.6827 (9)
Ca1—F3^i^	2.4105 (9)	Si1—F3	1.6942 (9)
Ca1—O1	2.4331 (13)		

Symmetry code: (i) 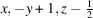 .

**Table 2:** Hydrogen-bond geometry (Å, °)

*D*—H⋯*A*	*D*—H	H⋯*A*	*D*⋯*A*	*D*—H⋯*A*
O1—H1⋯F3^ii^	0.78 (3)	2.19 (3)	2.9042 (14)	153 (3)
O1—H2*B*⋯O1^iii^	0.90 (5)	1.98 (5)	2.856 (3)	167 (4)
O1—H2*A*⋯O1^iv^	0.77 (5)	2.17 (5)	2.902 (3)	159 (4)

Symmetry codes: (ii) 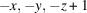 ; (iii) 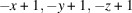 ; (iv) 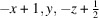 .

**Table 3:** Crystal structures used for the calculation of the new empirical *R*_0_ bond-valence parameter for Si^IV^–F

Compound	ICSD number	Reference	Si—F bond-length range (Å)	BVS for Si (*R*_0_ from Brese & O’Keeffe, 1991 ▸)	BVS for Si (new *R*_0_)
BaSiF_6_	60882	(Svensson *et al.*, 1986 ▸)	1.688 (2)	4.481	3.968
(CH_3_NH_3_)_2_SiF_6_	110673	(Conley *et al.*, 2002 ▸)	1.6810 (12)–1.6828 (17)	4.559	4.037
(CH_7_N_4_)_2_SiF_6_·2H_2_O	280103	(Ross *et al.*, 1999 ▸)	1.6797 (9)–1.6808 (9)	4.578	4.054
(CH_8_N_4_)SiF_6_	280102	(Ross *et al.*, 1999 ▸)	1.6684 (9)–1.7043 (9)	4.529	4.010
(C(NH_2_)_2_OH)_2_SiF_6_	63069	(Gubin *et al.*, 1988 ▸)	1.677 (2)–1.6971 (18)	4.513	3.996
(C(NH_2_)_3_)_2_SiF_6_	59237	(Waskowska, 1997 ▸)	1.6805 (12)–1.6833 (8)	4.550	4.029
(C_4_H_13_N_5_)SiF_6_	166449	(Gel’mbol’dt *et al.*, 2009 ▸)	1.657 (3)–1.698 (3)	4.643	4.111
CaSiF_6_·2H_2_O(*mP*2)	Present work		1.6808 (9)–1.6942 (9)	4.507	3.991
[Co(NH_3_)_5_(NO_2_)]SiF_6_	280030	(Naumov *et al.*, 1999 ▸)	1.6769 (18)–1.6899 (13)	4.495	3.981
CrSiF_6_·4H_2_O	165384	(Cotton *et al.*, 1993 ▸)	1.6640 (8)–1.6968 (8)	4.546	4.026
CsLiSiF_6_	142874	(Stoll *et al.*, 2021 ▸)	1.667 (2)–1.699 (2)	4.479	3.966
[Cu(bpy)_2_(H_2_O)]SiF_6_·4H_2_O	133607	(Nisbet *et al.*, 2021 ▸)	1.6677 (10)–1.6947 (9)	4.574	4.050
[Cu{SC(NH_2_)_2_}_4_]_2_SiF_6_	249750	(Bowmaker *et al.*, 2008 ▸)	1.663 (2)–1.696 (2)	4.585	4.060
CuSiF_6_·4H_2_O	165385	(Cotton *et al.*, 1993 ▸)	1.6686 (8)–1.6973 (9)	4.510	3.993
CuSiF_6_·6H_2_O	34760	(Ray *et al.*, 1973*b* ▸)	1.679 (5)	4.591	4.066
			1.659 (6)–1.674 (6)	4.765	4.219
H_2_SiF_6_·4H_2_O	40388	(Mootz & Oellers, 1988 ▸)	1.666 (1)–1.696 (1)	4.553	4.031
H_2_SiF_6_·6H_2_O	40389	(Mootz & Oellers, 1988 ▸)	1.677 (1)–1.704 (1)	4.447	3.938
H_2_SiF_6_·9.5H_2_O	40390	(Mootz & Oellers, 1988 ▸)	1.680 (1)–1.697 (1)	4.454	3.944
			1.684 (1)–1.706 (1)	4.448	3.939
K_2_SiF_6_(*cF*4)	420429	(Kutoglu *et al.*, 2009 ▸)	1.6873 (16)	4.490	3.975
K_2_SiF_6_(*hP*2)	158483	(Gramaccioli & Campostrini, 2007 ▸)	1.681 (2)–1.689 (2)	4.518	4.000
K_2_SiF_6_·KNO_3_	417735	(Rissom *et al.*, 2008 ▸)	1.6782 (6)	4.601	4.074
KLiSiF_6_	142875	(Stoll *et al.*, 2021 ▸)	1.676 (1)–1.701 (1)	4.495	3.980
KNaSiF_6_	71334	(Fischer & Krämer, 1991 ▸)	1.641 (5)–1.678 (5)	4.860	4.304
K_3_Na(SiF_6_)(TaF_7_)	122403	(Tang *et al.*, 2021 ▸)	1.665 (3)–1.702 (3)	4.558	4.036
K_3_Na_4_(BF_4_)(SiF_6_)_3_	121301	(Bandemehr *et al.*, 2020 ▸)	1.650 (2)–1.699 (2)	4.535	4.015
			1.666 (2)–1.700 (1)	4.560	4.038
Li_2_SiF_6_	425923	(Hinter­egger *et al.*, 2014 ▸)	1.685 (2)	4.518	4.000
			1.690 (2)–1.690 (8)	4.457	3.947
MgSiF_6_·6H_2_O	250196	(Cherkasova *et al.*, 2004 ▸)	1.6888 (9)–1.7465 (10)	4.194	3.714
MnSiF_6_·6H_2_O	59274	(Torii *et al.*, 1997 ▸)	1.690 (7)	4.457	3.947
			1.668 (7)–1.693 (7)	4.575	4.051
(NH_3_OH)_2_SiF_6_·2H_2_O	94567	(Kristl *et al.*, 2002 ▸)	1.6793 (10)–1.6837 (10)	4.570	4.046
(NH_4_)_2_SiF_6_	54724	(Fábry *et al.*, 2001 ▸)	1.695 (1)–1.700 (1)	4.368	3.867
(N_2_H_5_)_2_SiF_6_	776	(Ouasri *et al.*, 2019 ▸)	1.6777 (4)–1.7101 (4)	4.476	3.963
(N_2_H_6_)SiF_6_	35702	(Cameron *et al.*, 1983 ▸)	1.671 (1)–1.683 (1)	4.596	4.070
Na_2_SiF_6_	433134	(Zhang *et al.*, 2017 ▸)	1.6755 (14)–1.6756 (14)	4.635	4.104
			1.6907 (16)–1.6916 (11)	4.443	3.934
PbSiF_6_·2H_2_O	39358	(Golubev *et al.*, 1991 ▸)	1.645 (10)–1.707 (10)	4.558	4.036
			1.664 (10)–1.716 (10)	4.411	3.906
Rb_2_SiF_6_	136303	(Rienmüller *et al.*, 2021 ▸)	1.693 (3)	4.421	3.915
[RuF(NH_3_)_4_(NO)]SiF_6_	703	(Mikhailov *et al.*, 2019 ▸)	1.661 (1)–1.713 (2)	4.556	4.035
[Ru_2_(H_2_O)_2_(NH_4_)_8_S_2_](SiF_6_)_2_	111446	(Woods & Wilson, 2021 ▸)	1.666 (2)–1.7065 (19)	4.552	4.031
SiF_4_	48147	(Mootz & Korte, 1984 ▸)	1.5401 (6)	4.455	3.945
SrSiF_6_·2H_2_O	20552	(Golovastikov & Belov, 1982 ▸)	1.675 (5)–1.700 (5)	4.502	3.987
[Tl_2_(NH_3_)_6_]SiF_6_·2NH_3_	144214	(Rudel *et al.*, 2021 ▸)	1.687 (2)–1.6877 (15)	4.488	3.974
Tl_2_SiF_6_	136300	(Rienmüller *et al.*, 2021 ▸)	1.686 (6)	4.505	3.989
Tl_3_F[SiF_6_]	136302	(Rienmüller *et al.*, 2021 ▸)	1.688 (6)–1.695 (6)	4.439	3.931

**Table 4:** Comparison of the BVS calculation results for Si^IV^ of crystal structures collected in Table 3 ▸ employing the new *R*_0_ parameter and the previously reported parameter

	*R*_0_	Maximum BVS	Minimum BVS	Mean BVS	Standard deviation	% of data within ± 0.2 v.u.	% of data within ± 0.1 v.u.
This study	1.534	4.304	3.714	4.005	0.086	93.9	87.8
Brese & O’Keeffe (1991 ▸)	1.58	4.860	4.194	4.522	0.098	2.0	0

**Table 5:** Experimental details

Crystal data	
Chemical formula	CaSiF_6_·2H_2_O
*M*_r_	218.20
Crystal system, space group	Monoclinic, *P*2/*c*
Temperature (K)	100
*a*, *b*, *c* (Å)	5.96605 (17), 5.13977 (12), 9.9308 (3)
β (°)	107.275 (3)
*V* (Å^3^)	290.78 (1)
*Z*	2
Radiation type	Cu *K*α
μ (mm^−1^)	12.29
Crystal size (mm)	0.15 × 0.08 × 0.02

Data collection	
Diffractometer	XtaLAB Synergy-S, Dualflex, Eiger2 R CdTe 1M
Absorption correction	Gaussian (*CrysAlis PRO*; Rigaku OD, 2022 ▸)
*T*_min_, *T*_max_	0.365, 1.000
No. of measured, independent and observed [*I* > 2σ(*I*)] reflections	8322, 608, 598
*R*_int_	0.051
(sin θ/λ)_max_ (Å^−1^)	0.628

Refinement	
*R*[*F* ^2^ > 2σ(*F* ^2^)], *wR*(*F* ^2^), *S*	0.025, 0.070, 1.14
No. of reflections	608
No. of parameters	61
H-atom treatment	All H-atom parameters refined
Δρ_max_, Δρ_min_ (e Å^−3^)	0.32, −0.37

Computer programs: *CrysAlis PRO* (Rigaku OD, 2022 ▸), *SUPERFLIP* (Palatinus & Chapuis, 2007 ▸), *SHELXL2019/2* (Sheldrick, 2015 ▸), *OLEX2* (Dolomanov *et al.*, 2009 ▸), *DIAMOND* (Brandenburg, 2005 ▸) and *publCIF* (Westrip, 2010 ▸).

## supplementary crystallographic information

### Crystal data

**Table:**

CaSiF_6_·2H_2_O	*F*(000) = 216
*M**_r_* = 218.20	*D*_x_ = 2.492 Mg m^−^^3^
Monoclinic, *P*2/*c*	Cu *K*α radiation, λ = 1.54184 Å
*a* = 5.96605 (17) Å	Cell parameters from 5902 reflections
*b* = 5.13977 (12) Å	θ = 7.8–75.3°
*c* = 9.9308 (3) Å	µ = 12.29 mm^−^^1^
β = 107.275 (3)°	*T* = 100 K
*V* = 290.78 (1) Å^3^	Plate, colourless
*Z* = 2	0.15 × 0.08 × 0.02 mm

### Data collection

**Table:**

XtaLAB Synergy-S, Dualflex, Eiger2 R CdTe 1M diffractometer	608 independent reflections
Radiation source: micro-focus sealed X-ray tube, PhotonJet (Cu) X-ray Source	598 reflections with *I* > 2σ(*I*)
Mirror monochromator	*R*_int_ = 0.051
Detector resolution: 13.3333 pixels mm^-1^	θ_max_ = 75.4°, θ_min_ = 7.8°
ω scans	*h* = −7→7
Absorption correction: gaussian (CrysalisPro; Rigaku OD, 2022)	*k* = −6→6
*T*_min_ = 0.365, *T*_max_ = 1.000	*l* = −12→12
8322 measured reflections	

### Refinement

**Table:**

Refinement on *F*^2^	Primary atom site location: iterative
Least-squares matrix: full	Hydrogen site location: difference Fourier map
*R*[*F*^2^ > 2σ(*F*^2^)] = 0.025	All H-atom parameters refined
*wR*(*F*^2^) = 0.070	*w* = 1/[σ^2^(*F*_o_^2^) + (0.0516*P*)^2^ + 0.0488*P*] where *P* = (*F*_o_^2^ + 2*F*_c_^2^)/3
*S* = 1.14	(Δ/σ)_max_ < 0.001
608 reflections	Δρ_max_ = 0.32 e Å^−^^3^
61 parameters	Δρ_min_ = −0.37 e Å^−^^3^
0 restraints	

### Special details

**Table:**

Geometry. All esds (except the esd in the dihedral angle between two l.s. planes) are estimated using the full covariance matrix. The cell esds are taken into account individually in the estimation of esds in distances, angles and torsion angles; correlations between esds in cell parameters are only used when they are defined by crystal symmetry. An approximate (isotropic) treatment of cell esds is used for estimating esds involving l.s. planes.

### Fractional atomic coordinates and isotropic or equivalent isotropic displacement parameters (Å^2^)

**Table:**

	*x*	*y*	*z*	*U*_iso_*/*U*_eq_	Occ. (<1)
Ca1	0.000000	0.58252 (7)	0.250000	0.01360 (19)	
Si1	0.000000	0.000000	0.500000	0.0135 (2)	
F1	−0.06447 (16)	0.26688 (17)	0.39793 (9)	0.0187 (3)	
F2	0.20424 (15)	0.17052 (18)	0.62125 (9)	0.0178 (2)	
F3	−0.19861 (16)	0.09149 (15)	0.58239 (10)	0.0164 (3)	
O1	0.3884 (2)	0.4033 (2)	0.36145 (15)	0.0190 (3)	
H1	0.378 (5)	0.255 (6)	0.373 (3)	0.035 (7)*	
H2B	0.469 (8)	0.483 (9)	0.441 (5)	0.016 (13)*	0.49 (5)
H2A	0.475 (8)	0.419 (7)	0.318 (5)	0.017 (13)*	0.51 (5)

### Atomic displacement parameters (Å^2^)

**Table:**

	*U*^11^	*U*^22^	*U*^33^	*U*^12^	*U*^13^	*U*^23^
Ca1	0.0176 (3)	0.0092 (3)	0.0144 (3)	0.000	0.00529 (18)	0.000
Si1	0.0181 (3)	0.0090 (3)	0.0140 (3)	0.0000 (2)	0.0059 (2)	0.0000 (2)
F1	0.0251 (5)	0.0124 (4)	0.0201 (5)	0.0025 (4)	0.0091 (4)	0.0041 (3)
F2	0.0189 (5)	0.0155 (4)	0.0190 (5)	−0.0010 (3)	0.0056 (4)	−0.0037 (3)
F3	0.0201 (5)	0.0120 (5)	0.0185 (5)	−0.0003 (3)	0.0080 (4)	−0.0020 (3)
O1	0.0199 (6)	0.0145 (6)	0.0224 (6)	−0.0008 (4)	0.0058 (5)	0.0006 (4)

### Geometric parameters (Å, º)

**Table:**

Ca1—Si1^i^	3.2815 (3)	Si1—F1^vi^	1.6808 (9)
Ca1—Si1^ii^	3.2815 (3)	Si1—F1	1.6809 (9)
Ca1—F1	2.2965 (9)	Si1—F2^vi^	1.6826 (9)
Ca1—F1^iii^	2.2965 (9)	Si1—F2	1.6827 (9)
Ca1—F2^iv^	2.3783 (9)	Si1—F3	1.6942 (9)
Ca1—F2^v^	2.3783 (9)	Si1—F3^vi^	1.6942 (9)
Ca1—F3^iv^	2.4105 (9)	O1—H1	0.78 (3)
Ca1—F3^v^	2.4105 (9)	O1—H2B	0.90 (5)
Ca1—O1	2.4331 (13)	O1—H2A	0.77 (5)
Ca1—O1^iii^	2.4331 (13)		
			
Si1^ii^—Ca1—Si1^i^	98.328 (10)	O1—Ca1—Si1^i^	112.20 (3)
F1^iii^—Ca1—Si1^i^	86.58 (2)	O1^iii^—Ca1—Si1^ii^	112.20 (3)
F1—Ca1—Si1^i^	169.60 (2)	O1^iii^—Ca1—Si1^i^	96.74 (3)
F1^iii^—Ca1—Si1^ii^	169.60 (2)	O1^iii^—Ca1—O1	135.49 (6)
F1—Ca1—Si1^ii^	86.58 (2)	Ca1^vii^—Si1—Ca1^v^	180.0
F1—Ca1—F1^iii^	90.11 (4)	F1—Si1—Ca1^v^	82.60 (3)
F1—Ca1—F2^iv^	158.57 (3)	F1—Si1—Ca1^vii^	97.40 (3)
F1—Ca1—F2^v^	79.82 (3)	F1^vi^—Si1—Ca1^v^	97.40 (3)
F1^iii^—Ca1—F2^iv^	79.82 (3)	F1^vi^—Si1—Ca1^vii^	82.60 (3)
F1^iii^—Ca1—F2^v^	158.57 (3)	F1^vi^—Si1—F1	180.0
F1—Ca1—F3^iv^	142.32 (3)	F1^vi^—Si1—F2^vi^	89.65 (5)
F1—Ca1—F3^v^	100.99 (3)	F1—Si1—F2^vi^	90.35 (5)
F1^iii^—Ca1—F3^v^	142.32 (3)	F1—Si1—F2	89.65 (5)
F1^iii^—Ca1—F3^iv^	100.99 (3)	F1^vi^—Si1—F2	90.35 (5)
F1—Ca1—O1	76.07 (4)	F1—Si1—F3	89.83 (4)
F1^iii^—Ca1—O1^iii^	76.07 (4)	F1—Si1—F3^vi^	90.17 (4)
F1^iii^—Ca1—O1	72.88 (4)	F1^vi^—Si1—F3	90.17 (4)
F1—Ca1—O1^iii^	72.88 (4)	F1^vi^—Si1—F3^vi^	89.83 (4)
F2^v^—Ca1—Si1^ii^	29.44 (2)	F2—Si1—Ca1^v^	44.00 (3)
F2^v^—Ca1—Si1^i^	99.95 (3)	F2—Si1—Ca1^vii^	136.00 (3)
F2^iv^—Ca1—Si1^i^	29.44 (2)	F2^vi^—Si1—Ca1^v^	136.00 (3)
F2^iv^—Ca1—Si1^ii^	99.95 (3)	F2^vi^—Si1—Ca1^vii^	44.00 (3)
F2^iv^—Ca1—F2^v^	115.49 (5)	F2^vi^—Si1—F2	180.0
F2^iv^—Ca1—F3^v^	77.00 (3)	F2—Si1—F3^vi^	91.63 (4)
F2^v^—Ca1—F3^v^	58.87 (3)	F2^vi^—Si1—F3	91.63 (4)
F2^v^—Ca1—F3^iv^	77.00 (3)	F2—Si1—F3	88.37 (4)
F2^iv^—Ca1—F3^iv^	58.87 (3)	F2^vi^—Si1—F3^vi^	88.37 (4)
F2^v^—Ca1—O1^iii^	82.87 (4)	F3—Si1—Ca1^v^	45.25 (3)
F2^iv^—Ca1—O1^iii^	121.89 (4)	F3^vi^—Si1—Ca1^v^	134.75 (3)
F2^iv^—Ca1—O1	82.87 (4)	F3—Si1—Ca1^vii^	134.75 (3)
F2^v^—Ca1—O1	121.89 (4)	F3^vi^—Si1—Ca1^vii^	45.25 (3)
F3^v^—Ca1—Si1^ii^	29.94 (2)	F3—Si1—F3^vi^	180.0
F3^v^—Ca1—Si1^i^	87.56 (2)	Si1—F1—Ca1	155.57 (5)
F3^iv^—Ca1—Si1^i^	29.94 (2)	Si1—F2—Ca1^v^	106.56 (4)
F3^iv^—Ca1—Si1^ii^	87.56 (2)	Si1—F3—Ca1^v^	104.81 (4)
F3^v^—Ca1—F3^iv^	91.93 (4)	Ca1—O1—H1	110 (2)
F3^v^—Ca1—O1	75.09 (4)	Ca1—O1—H2B	115 (3)
F3^v^—Ca1—O1^iii^	141.61 (4)	Ca1—O1—H2A	114 (3)
F3^iv^—Ca1—O1	141.61 (4)	H1—O1—H2B	111 (3)
F3^iv^—Ca1—O1^iii^	75.09 (4)	H1—O1—H2A	107 (3)
O1—Ca1—Si1^ii^	96.74 (3)		
			
Ca1^v^—Si1—F1—Ca1	115.56 (12)	F2^vi^—Si1—F1—Ca1	−108.01 (13)
Ca1^vii^—Si1—F1—Ca1	−64.44 (12)	F2—Si1—F1—Ca1	71.99 (13)
Ca1^vii^—Si1—F2—Ca1^v^	180.000 (1)	F2^vi^—Si1—F3—Ca1^v^	−170.07 (5)
Ca1^vii^—Si1—F3—Ca1^v^	180.000 (1)	F2—Si1—F3—Ca1^v^	9.93 (5)
F1^vi^—Si1—F2—Ca1^v^	−100.32 (4)	F3^vi^—Si1—F1—Ca1	−19.64 (13)
F1—Si1—F2—Ca1^v^	79.69 (4)	F3—Si1—F1—Ca1	160.36 (13)
F1—Si1—F3—Ca1^v^	−79.72 (4)	F3^vi^—Si1—F2—Ca1^v^	169.84 (5)
F1^vi^—Si1—F3—Ca1^v^	100.28 (4)	F3—Si1—F2—Ca1^v^	−10.16 (5)

Symmetry codes: (i) −*x*, *y*+1, −*z*+1/2; (ii) *x*, *y*+1, *z*; (iii) −*x*, *y*, −*z*+1/2; (iv) *x*, −*y*+1, *z*−1/2; (v) −*x*, −*y*+1, −*z*+1; (vi) −*x*, −*y*, −*z*+1; (vii) *x*, *y*−1, *z*.

### Hydrogen-bond geometry (Å, º)

**Table:**

*D*—H···*A*	*D*—H	H···*A*	*D*···*A*	*D*—H···*A*
O1—H1···F3^vi^	0.78 (3)	2.19 (3)	2.9042 (14)	153 (3)
O1—H2*B*···O1^viii^	0.90 (5)	1.98 (5)	2.856 (3)	167 (4)
O1—H2*A*···O1^ix^	0.77 (5)	2.17 (5)	2.902 (3)	159 (4)

Symmetry codes: (vi) −*x*, −*y*, −*z*+1; (viii) −*x*+1, −*y*+1, −*z*+1; (ix) −*x*+1, *y*, −*z*+1/2.
